# Simulated rRNA/DNA Ratios Show Potential To Misclassify Active Populations as Dormant

**DOI:** 10.1128/AEM.00696-17

**Published:** 2017-05-17

**Authors:** Blaire Steven, Cedar Hesse, John Soghigian, La Verne Gallegos-Graves, John Dunbar

**Affiliations:** a Department of Environmental Sciences, Connecticut Agricultural Experiment Station, New Haven, Connecticut, USA; b Bioscience Division, Los Alamos National Laboratory, Los Alamos, New Mexico, USA; University of Tennessee and Oak Ridge National Laboratory

**Keywords:** RNA/DNA ratios, activity, dormancy, error, sampling, simulation

## Abstract

The use of rRNA/DNA ratios derived from surveys of rRNA sequences in RNA and DNA extracts is an appealing but poorly validated approach to infer the activity status of environmental microbes. To improve the interpretation of rRNA/DNA ratios, we performed simulations to investigate the effects of community structure, rRNA amplification, and sampling depth on the accuracy of rRNA/DNA ratios in classifying bacterial populations as “active” or “dormant.” Community structure was an insignificant factor. In contrast, the extent of rRNA amplification that occurs as cells transition from dormant to growing had a significant effect (*P* < 0.0001) on classification accuracy, with misclassification errors ranging from 16 to 28%, depending on the rRNA amplification model. The error rate increased to 47% when communities included a mixture of rRNA amplification models, but most of the inflated error was false negatives (i.e., active populations misclassified as dormant). Sampling depth also affected error rates (*P* < 0.001). Inadequate sampling depth produced various artifacts that are characteristic of rRNA/DNA ratios generated from real communities. These data show important constraints on the use of rRNA/DNA ratios to infer activity status. Whereas classification of populations as active based on rRNA/DNA ratios appears generally valid, classification of populations as dormant is potentially far less accurate.

**IMPORTANCE** The rRNA/DNA ratio approach is appealing because it extracts an extra layer of information from high-throughput DNA sequencing data, offering a means to determine not only the seedbank of taxa present in communities but also the subset of taxa that are metabolically active. This study provides crucial insights into the use of rRNA/DNA ratios to infer the activity status of microbial taxa in complex communities. Our study shows that the approach may not be as robust as previously supposed, particularly in complex communities composed of populations employing different growth strategies, and identifies factors that inflate the erroneous classification of active populations as dormant.

## INTRODUCTION

Studies of microbial community composition are increasingly concerned with identifying metabolically active populations linked to ecosystem processes. Although amplification and sequencing of rRNA genes effectively document the microorganisms present in a community (1–4), the abundance of an organism in a community DNA survey is an unreliable indicator of its activity. Given the frequent presence of starving cells, spores, or other dormant forms of cells (5–8), nonviable cells with residual DNA (9), or extracellular DNA (10, 11), only a portion of the microbial DNA detected in the environment represents metabolically active cells (12). To identify the metabolically active members of microbial communities, sequencing of rRNA has been employed. Because rRNA is a structural component of ribosomes and ribosomes are expected to increase with metabolic activity (13–16), rRNA abundance is a potential marker of growth and metabolism. Additionally, RNA generally degrades faster than DNA after cell death, so detection of rRNA is more likely to indicate active or recently living cells (17).

Different approaches have been used to characterize active microbial populations via rRNA sequencing. One approach is to characterize only the rRNA pool and infer that this represents active populations (18, 19). However, the presence of rRNA is not always a reliable indicator of activity because dormant forms of cells harbor ribosomes in order to resume protein synthesis when environmental conditions improve (20). Comparison of rRNA and DNA quantities is a more sophisticated approach. As cellular activity increases, the ratio of rRNA to rRNA genes is expected to increase because ribosome abundance increases much more than the genome copy number in active cells. Consequently, organisms with a higher abundance of rRNA than DNA in community surveys are proposed to be active (21, 22). A critical detail of the rRNA/DNA method used in microbial community analyses is that the true ratios of rRNA to DNA occurring within cells are not measured; instead, the relative abundance of a taxon in a community survey of rRNA is compared to its relative abundance in a community survey of *rrn* genes. Although a number of studies have used rRNA/DNA ratios to characterize active microbial populations in environmental samples (23–25), there has been little effort to investigate factors that may affect data interpretation.

In this study, we performed simulations to test the effects of community structure, variation in rRNA amplification, and sampling depth on the identification of active populations on the basis of rRNA/DNA ratios. The simulation results were used to guide the interpretation of empirical sequence data generated from forest floor microbial communities. The simulation data revised our original interpretation of the empirical data, demonstrating the potential of these simulations to inform other studies that employ rRNA/DNA ratios.

## RESULTS

### rRNA amplification data.

**FIG 1:**
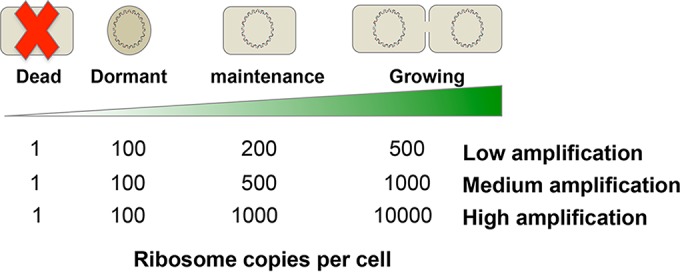
rRNA amplification and random assignment of cells to metabolic states. Three different rRNA amplification models were used to represent variation in the abundance of ribosomes in cells in four metabolic states. The number of ribosomes per cell in each of the activity metabolic states with the different rRNA amplification models is indicated. Data for the rRNA amplification models were based on data from Table S1. For simulations, each population in a community represented a mixture of cells in different metabolic states. The number of cells in a given population was determined from a community structure model. The cells in a population were randomly assigned to four metabolic states, and the net activity status of each population was calculated.

It is well accepted that the number of ribosomes (and therefore rRNA) in cells increases with the growth rate (26). Yet, the precise number of ribosomes in cells in different metabolic states is very uncertain. Among 18 studies published between 1986 and 2013, the quantity of ribosomes reported in bacterial cells varied 3,600-fold (see Table S1 in the supplemental material). The median quantity in stationary-phase cells was 200 (*n* = 3 studies; range, 20 to 8,000), and the median quantity in growing cells was about 5,100 (*n* = 18 studies and 13 species; range, 92 to 72,000). Given the uncertainty and large spread of published estimates, we defined three rRNA amplification models—low, medium, and high—to represent a range of possibilities for the increase in the ribosome content of cells across four metabolic states (Fig. 1). Among the three models, the cellular ribosome content ranged from 1 in dead cells to a maximum of 10,000 in growing cells.

### Effects of community structure and rRNA amplification model on rRNA/DNA ratios.

Simulations of communities with 5,000 populations showed that community structure did not have a significant effect on the accuracy of population activity assessments derived from rRNA/DNA ratios (by analysis of variance [ANOVA], degree of freedom [df] = 2, *F* = 0.002, and *P* = 0.99) (Table 1). These results were obtained from simulations (100 runs each) that represented three community structures and the three ribosome amplification models described in Fig. 1. Given a constant rRNA amplification model, the false detection rate (false positives [FP] plus false negatives [FN]) with different community structures was nearly identical (Table 1). For example, the false detection rates ranged from 21.3 to 21.5% for three community structures with the low rRNA amplification model (Table 1; similar results [not shown] were obtained from simulations with 1,000 runs). The data suggest that dramatic differences in community structure would not undermine the use of rRNA/DNA ratios to infer population activity status.

**TABLE 1:** Effect of community structure and ribosomal amplification on misclassifications

Ribosomal amplification	Community structure	Avg % FP ± SD^*a*^	Avg % FN ± SD^*a*^
Low	Lognormal σ1	10.7 ± 1.9	10.8 ± 1.9
Low	Lognormal σ2	10.7 ± 0.6	10.6 ± 0.6
Low	Even	10.7 ± 0.5	10.6 ± 0.4
Medium	Lognormal σ1	8.4 ± 1.8	8.1 ± 1.8
Medium	Lognormal σ2	8.1 ± 0.7	8.1 ± 0.6
Medium	Even	8.0 ± 0.5	8.0 ± 0.6
High	Lognormal σ1	13.9 ± 1.5	14.1 ± 1.8
High	Lognormal σ2	14.0 ± 0.6	13.9 ± 0.6
High	Even	13.9 ± 0.5	14.0 ± 0.6

a Percentages of the 5,000 input populations in 100 independent runs per simulation are shown.

**FIG 2:**
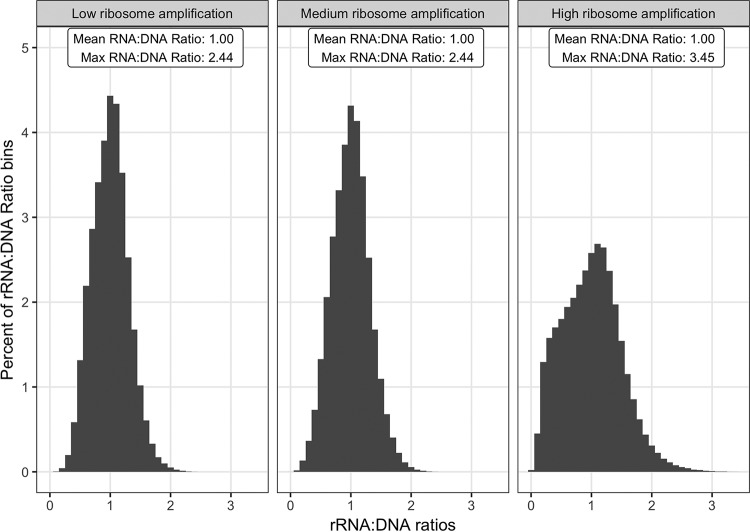
Effects of rRNA amplification models on rRNA/DNA ratios. Shown are histograms of rRNA/DNA ratios for each population generated from the simulations. The simulations represent the low, medium, and high rRNA amplification models run under the lognormal σ1 community structure model. Qualitatively similar data were obtained with the σ0 and σ2 community structure models, so the data are not shown. Each histogram represents the cumulative data of 100 independent runs of a simulation.

In contrast, rRNA amplification models had a significant effect on false detection rates (by ANOVA, df = 2, *F* = 3,869, and *P* < 0.0001). The combined FP and FN rates ranged from 16 to 28%, and the highest rate occurred with the high rRNA amplification model. The distribution of rRNA/DNA ratios in these simulations is illustrated in Fig. 2. The mean of the rRNA/DNA ratios was 1, but the mode shifted from 1 to 1.1 under the high rRNA amplification model. Similarly, the maximum rRNA/DNA ratio increased from ca. 2.4 in the low and medium models to 3.5 in the high rRNA amplification model.

Taken together, these data show that rRNA/DNA ratios are insensitive to variation in community structure, whereas variation in evolved growth strategy (i.e., rRNA amplification) among populations or communities can substantially influence the interpretation of rRNA/DNA ratios.

### Effect of partial sampling on rRNA/DNA ratios.

**FIG 3:**
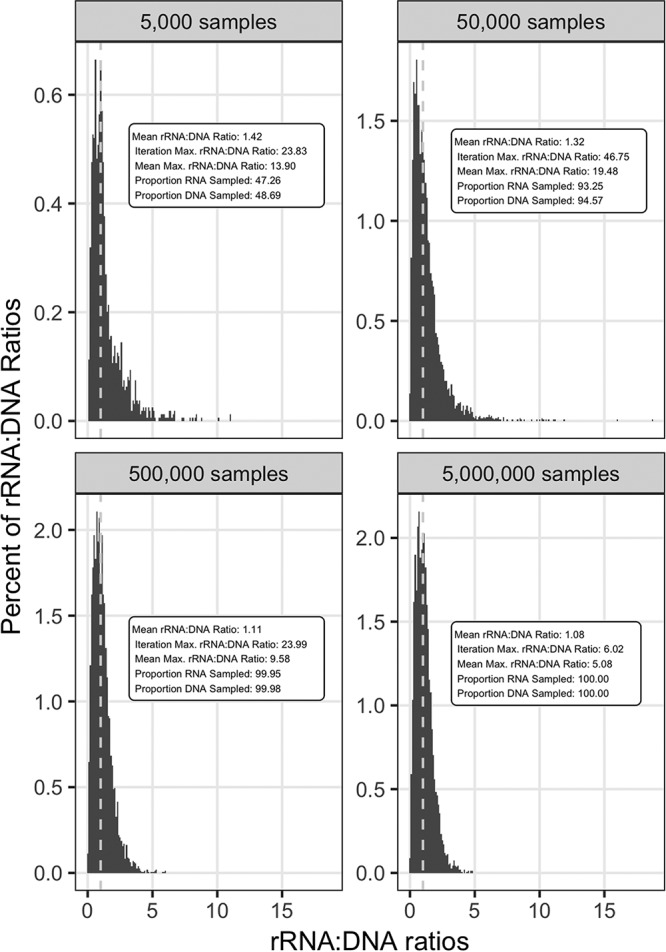
Effects of partial sampling on rRNA/DNA ratios. Each panel represents a different sampling depth, ranging from random samples of 1 to 1,000 times the input population richness. The simulation data presented are for a lognormal σ1 community structure with the medium rRNA amplification model and 5,000 populations. Data from other model permutations were qualitatively similar (data not shown). Each histogram represents illustrative data from a single run of a simulation. Summary statistics of combined data from 100 independent runs are included in the insets. The reported proportions of RNA and DNA sampled refer to the average proportions of the 5,000 input taxa that were detected in the samples.

Simulations with partial sampling—analogous to incomplete sampling of natural communities—showed that sampling depth had a significant effect on misclassification rates (Table 2) (by ANOVA, df = 4, *F* = 17.65, and *P* < 0.0001; similar results [not shown] were obtained from simulations with 1,000 runs). In these simulations, a community with a lognormal population distribution (standard deviation of 1) was used and random samples of increasing size were drawn (Fig. 3; Table 2). The simulations showed two major effects of sampling depth. First, undersampling of the communities produced a spread of rRNA/DNA ratios much greater than 1.0. For example, in simulations with the medium rRNA amplification model, rRNA/DNA ratios as high as 46.8 occurred when the sampling depth was 1 to 100 times the total number of populations (5,000) in the community (Fig. 3). At a sampling depth of 1,000×, the maximum rRNA/DNA ratio was only 6.0 (Fig. 3) and the distribution of rRNA/DNA ratios began to resemble the distribution in completely sampled communities (Fig. 2, middle panel) that had a maximum rRNA/DNA ratio of 2.4. Second, undersampling significantly inflated the misclassification rate for activity status (by ANOVA, *P* < 0.001), and the degree of this effect depended significantly on the rRNA amplification model (by ANOVA, df = 2, *F* = 10.85, and *P* = 0.005). Moreover, partial sampling increased the FN rate compared to the FP rate (by paired-sample two-tailed Student *t* test, *P* = 0.006). Increasing the sampling depth from 1× to 10,000× the total population richness reduced the combined FN and FP rate from an average of 38% to an average of 22% (Table 2), and the latter level was similar to levels observed in completely sampled communities (Table 1). These findings show that sampling depth is an important concern when using rRNA/DNA ratios to infer the activity of microbial populations.

**TABLE 2:** Effect of sample size on misclassifications

Sample size	Ribosomal amplification	Avg % FP ± SD^*a*^	Avg % FN ± SD^*a*^
5,000 (1×)^*b*^	Low	18.4 ± 0.6	20.2 ± 0.7
5,000 (1×)	Medium	17.3 ± 0.7	19.4 ± 0.7
5,000 (1×)	High	17.8 ± 0.7	20.4 ± 0.7
50,000 (10×)	Low	14.7 ± 0.6	16.2 ± 0.6
50,000 (10×)	Medium	13.2 ± 0.6	14.5 ± 0.6
50,000 (10×)	High	15.3 ± 0.6	16.9 ± 0.6
500,000 (100×)	Low	14.1 ± 0.6	14.5 ± 0.7
500,000 (100×)	Medium	9.2 ± 0.6)	9.8 ± 0.5
500,000 (100×)	High	14.0 ± 0.6	14.7 ± 0.6
5,000,000 (1,000×)	Low	10.8 ± 0.7	10.8 ± 0.6
5,000,000 (1,000×)	Medium	8.3 ± 0.6	8.4 ± 0.6
5,000,000 (1,000×)	High	14.1 ± 0.7	14.1 ± 0.7
50,000,000 (10,000×)	Low	10.7 ± 0.6	10.5 ± 0.7
50,000,000 (10,000×)	Medium	8.1 ± 0.6	8.1 ± 0.6
50,000,000 (10,000×)	High	14.0 ± 0.6	14.0 ± 0.7

a The error rates are expressed as percentages of the populations that were detected in both DNA and RNA sampling profiles, and each value is the average of 100 independent iterations ± the standard deviation). With low sampling depths, the number of detected populations was less than the 5,000 input populations.

b The parenthetical factors indicate the sampling depth as a multiple of the number of input populations (5,000) in the simulated community.

### Effect of mixed rRNA amplification on rRNA/DNA ratios.

Given that species have evolved different growth strategies, rRNA amplification is expected to vary among populations in natural communities. For example, a species with an inherently low growth rate is expected to have a lower number of ribosomes per cell (i.e., low ribosomal amplification) than a species with an inherently high growth rate when both species are in the active growth phase (15). To evaluate this effect, we constructed communities with a mixture of three rRNA amplification models. In these simulations, populations were randomly assigned to one of the three rRNA amplification models, yielding a community in which populations can have identical activity levels (i.e., they are equally active) but different ribosome counts. These simulations showed a dramatic increase in the total misclassification rate (47% versus 16 to 28%) of population activity status from rRNA/DNA ratios in comparison to simulations with a single rRNA amplification model (Table 3 versus Table 1). The increased error rate arose mainly from an increase in the FN rate to 34%, which was more than twice the 13% FP rate from a single rRNA amplification model (Table 3). When the populations that comprised the FP and FN detections were identified, 100% of the FP populations had the high rRNA amplification model, whereas only 1.4% of the FN populations harbored the high amplification model (Table 3). Conversely, ca. 99% of the FN populations were from the low and medium rRNA amplification models. These results suggest that a high FN rate is a likely characteristic of rRNA/DNA ratios from natural communities where growth strategies vary among populations, and therefore, “dormant” status may often be an incorrect inference. In contrast, the majority of populations classified as “active” from rRNA/DNA ratios are likely to be valid, but “active” status may be biased toward species with high rRNA amplification growth strategies.

**TABLE 3:** Effect of a mixed community on misclassifications

Error	% of total misclassification^*a*^	% of error from ribosome amplification model^*b*^		
		Low	Medium	High
FP	12.8	0	0	100
FN	33.8	49.2	49.4	1.4

a Percentage of misclassifications in the entire community (5,000 populations, 100 runs per simulation).

b Percent contributions of the different amplification models to the FP and FN error rates.

### Empirical sequencing data and rRNA/DNA ratios.

**FIG 4:**
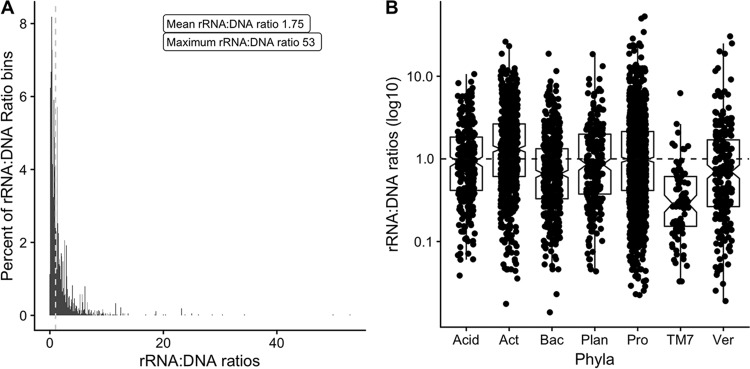
rRNA/DNA ratios in empirical sequence data. (A) Histogram of the ratios of OTUs from forest floor sequence libraries. Ratios were calculated individually for every OTU from 12 paired replicate sequence libraries. (B) Relationship between rRNA/DNA ratios and taxonomy. Each point represents an individual OTU. Only phyla that were present across all of the sequence libraries and accounted for >5% of the sequences are displayed. Given the large range of rRNA/DNA ratios, the *y* axis is displayed as a log scale. The dashed line represents an rRNA/DNA ratio of 1. OTUs above the line would be classified as active, and those below the line would be classified as dormant. The boxes denote the interquartile range of the rRNA/DNA ratios, with the notch denoting the mean. Phylum abbreviations: Acid, Acidobacteria; Act, Actinobacteria; Bac, Bacteroidetes; Plan, Planctomycetes; Pro, Proteobacteria; Ver, Verrucomicrobia.

rRNA/DNA ratios were calculated for operational taxonomic units (OTUs) found in each of 12 forest floor soil communities that were sampled from four sites across a 500-km transect in Michigan, USA. The sequence surveys represented only a portion of the total diversity of the samples, as indicated by Good's coverage (27), which ranged from ca. 61 to 70% across the sequence libraries. The rRNA/DNA ratios calculated from the surveys ranged from 0.003 to 53 (Fig. 4A). Qualitatively, the distribution of rRNA/DNA ratios appeared similar to the distributions from undersampled communities *in silico* (Fig. 3).

To investigate possible relationships between taxonomy and rRNA/DNA ratios, every OTU in the empirical sequencing data was assigned to a phylum level taxonomic bin. The rRNA/DNA ratios within each of the seven numerically dominant phyla (defined as those consistently present at >5% abundance in the sequence libraries) are presented in Fig. 4B. In this analysis, OTUs within the phyla Acidobacteria, Actinobacteria, Bacteroidetes, and Proteobacteria mostly had mean rRNA/DNA ratios close to or equal to 1.0, whereas the rRNA/DNA ratios of OTUs within the phylum TM7 (Candidatus Saccharibacteria [28]) were almost exclusively <1.0 (Fig. 4B).

## DISCUSSION

Our simulations verified that rRNA/DNA ratios can identify active populations but false detections can occur. False detection rates increased as the sampling depth decreased (Table 2). More importantly, the false detection rate was dramatically higher (48% versus 16 to 28%) when communities had populations that differed in rRNA amplification (Table 3). We did not test the combined effect of undersampling and mixed rRNA amplification, but the combined factors are likely to inflate false detection rates even more. Given the high diversity of most terrestrial microbial communities and the range of physiologies and growth rates among populations, high false detection rates are anticipated when rRNA/DNA ratios are used to infer activity status in complex environmental samples. The strong bias toward inflation of FN over FP (Tables 2 and 3) shows that classification of populations as “dormant” is less reliable than classification as “active.”

We expected the misclassification error to be higher in communities with a strong dominance pattern—that is, a less even species abundance distribution—but simulations did not support this hypothesis. The logic of our hypothesis was that increased dominance would exacerbate measurement errors in the RNA or DNA abundance of rare types, giving rise to greatly distorted rRNA/DNA ratios of rare types. Why? Because a statistical characteristic of community survey data is that the relative measurement error of taxon abundance is greater for rare taxa than for abundant taxa in community surveys. We can only speculate that a community structure effect was not observed because the misclassification of activity status depends foremost on the distribution of activity levels among species, not species abundance. Our simulations used a normal distribution of activity levels, owing to the representation of each taxon as a random mixture of four metabolic states. This is an important caveat for future studies: a different distribution of activity levels may produce different misclassification error rates.

Given the propensity for FN to occur with rRNA/DNA ratios from complex communities (Tables 2 and 3), inferring the dormancy of a specific taxon in natural communities should be done with caution. In our forest floor samples, OTUs within the phylum TM7 almost exclusively showed rRNA/DNA ratios of <1 (Fig. 4B), possibly indicating consistent dormancy along the 500-km transect we sampled. Our simulations, however, suggest the alternative explanation that TM7 bacteria may simply be more prone to misclassification as FN. This phylum may be misclassified as dormant if it has evolved a low rRNA amplification strategy, whereas co-occurring active taxa have medium to high rRNA amplification (Table 3). This simulation-informed insight demonstrates the value of using modeling to guide the interpretation of empirical data, especially when a foundation of empirical ground truth is absent.

The findings from simulations improve the interpretation of rRNA/DNA ratios of natural communities. A characteristic feature of rRNA/DNA ratios derived from our natural communities and found in other empirical studies is a wide spread of ratios much greater than 1. The highest ratio we observed was 53 (Fig. 4A), and ratios of up to 122 were reported in aerosol samples (29). Although it is tempting to move beyond the classification of taxa as active or dormant and use rRNA/DNA ratios to distinguish degrees of activity (also known as “specific activity” [29]), several considerations suggest that the practice is generally unsound. First, insufficient sampling depth can dramatically distort rRNA/DNA ratios, as our simulations showed (Fig. 3). Second, for some species, rRNA/DNA ratios are misleading indicators of their general activity status (active versus dormant), much less the degree of activity, because of eccentric biology. For example, the cyanobacterium Aphanizomenon ovalisporum forms dormant cells called akinetes that contain a greater abundance of ribosomes than vegetative cells do (30), and among members of the genus Bacillus, high ribosome content may be a prerequisite to enter dormancy (20). Finally, the species-specific interplay among genomic factors (discussed below), ribosome content, and growth strategy makes fine-scale interpretation of rRNA/DNA ratios difficult. The scaling of rRNA/DNA ratios with specific activity levels is likely to vary considerably among species, such that two species with the same rRNA/DNA ratio can have different activity levels or vice versa. Recently, Blazewicz et al. (31) reviewed various biological factors that affect the use of rRNA as a general indicator of activity. Our simulations showed that both methodological and biological factors can confound the use of rRNA/DNA ratios to infer specific activity.

Our simulations captured the effects of several biological factors that could contribute to rRNA and rRNA gene counts in natural communities. The use of different community structures captured variation in rRNA gene abundance among populations that may arise not only from differences in cellular abundance but also from several genomic factors, namely, variation in the copy number of *rrn* genes within genomes, genome ploidy, and growth rate. In bacteria, rRNA gene copy numbers vary from 1 to as many as 15 per genome (32, 33) and some species of bacteria are polyploids that harbor multiple copies of their genome (34, 35). “Epulopiscium” sp. is an extreme polyploid case with thousands of copies of the genome per cell during normal growth (36). The copy number of rRNA genes per cell can also increase transiently as the growth rate increases owing to the presence of multiple genome replication forks per cell (37, 38). All of these factors contribute to the observed abundance distribution of rRNA gene sequences—the community structure—in targeted metagenomic surveys of natural communities.

Genome factors and other regulatory phenomena also contribute to differences among species in growth strategy—a multifaceted evolutionary trait that includes how species manage ribosome content (rRNA abundance). Our use of three different rRNA amplification models reflected the uncertainty in the current state of knowledge concerning the ribosome content of cells in different physiological states, as well as the variation in growth strategy among species in natural communities. Detailed information about rRNA amplification among species with different growth strategies is a large knowledge gap that merits further attention and careful physiological studies.

Given our observations and the paucity of data on the growth strategies of most bacterial species, the safest application of rRNA/DNA ratios may be time series studies, in which the activity of a population is assessed relative to an initial baseline instead of relative to an assumption of a universal (across microbial phyla) scaling of rRNA/DNA ratios with population activity status. A substantial change in the rRNA/DNA ratio of a taxon can indicate a shift in activity level, reducing dependence on a universal threshold value (e.g., 1.0) for classification of populations as active or dormant. Time series analysis also mitigates the problem of legacy effects, wherein an rRNA/DNA point measurement reflects a prior activity status and fails to reveal populations that may be transitioning to a new activity status (12).

### Conclusion.

With a modest simulation effort, we gained important insights into the interpretation of the rRNA/DNA ratios of real communities. Our study showed that highly asymmetric FP and FN error rates are possible when rRNA/DNA ratios are applied to complex communities to infer population activity status. Inference of active status appears generally sound, whereas inference of dormant status is undermined by a potentially high misclassification error rate. Insufficient sampling depth and evolved physiological variation among species significantly inflate the FN rate (i.e., active populations misclassified as dormant). Validation of rRNA/DNA ratios with orthogonal techniques to measure population activity, such as stable isotope probing (39) or nanoscale secondary ion mass spectrometry (40), would improve the interpretation of metabolic activity. Finally, additional physiological studies that accurately characterize the relationship between ribosome content and specific growth rates, or activity status, among different species would also improve the modeling and interpretation of rRNA/DNA ratios.

## MATERIALS AND METHODS

### Simulations.

Simulations and statistical analyses were performed with the R software package (41). Every simulation represented a community made up of cells belonging to 5,000 populations. Each simulation involved three parameters, described below.

### Community structure (i.e., abundance distribution of populations).

Three community structures were tested. The community structures differed in the extent of population dominance, with dominance ranging from high to none. The community structures were as follows: a lognormal distribution with a standard deviation of 2, a lognormal distribution with a standard deviation of 1, and a uniform distribution with a standard deviation of 0 (i.e., equal abundance of all populations). These population abundance distributions were used to represent the abundance of cells (and *rrn* gene abundance) in each population.

### Metabolic states.

Each population was represented as a collection of cells in different metabolic states. This approach was used because microbial populations in natural environments are seldom expected to be metabolically homogeneous, owing to factors such as fluctuating and patchy resource distributions and complex ecological gradients created by nonuniform distributions of other interacting populations. For modeling, each cell in a population was assigned to one of four metabolic states—dead, dormant, maintenance, or growing (Fig. 1). The key characteristics of the metabolic states are as follows: (i) dead, cells contain DNA but very limited rRNA; (ii) dormant, cells have low ribosome numbers but are capable of resuscitation (e.g., spores); (iii) maintenance, cells are alive and metabolically active but maintain a low metabolic rate with little, if any, reproduction; (iv) growing, cells have high activity and are multiplying. The four metabolic states were based on those proposed by Blazewicz et al. (31). For each population, the fraction of cells in each metabolic state was assigned randomly, with the constraint that the sum of the fractions equals 1 (Fig. 1). Random assignment of the metabolic states within each population created an approximately normal distribution of activity levels among the 5,000 populations in each community.

### rRNA amplification.

We created three rRNA amplification models describing how ribosome abundance changes among the four cellular metabolic states (Fig. 1A). We derived the models on the basis of data from studies examining ribosome abundance in stationary-phase or growing cells of 13 bacterial species (see Table S1). Given a specific rRNA amplification model and the fractions of a population in the four metabolic states, the rRNA count of a population could be explicitly calculated as the sum of ribosomes in cells in the four metabolic states. The resulting relative abundance of rRNA of each population in a community could then be compared with the relative *rrn* gene abundance from the community structure model to calculate the rRNA/DNA ratio. Populations with an rRNA/DNA ratio of >1 were classified as active, and those with an rRNA/DNA ratio of <1 were classified as dormant, as in a previous study (21).

The simulations consisted of the following seven sequential steps. (i) Assign a community structure. (ii) Randomly assign cells within each population to four metabolic states. (iii) Select an rRNA amplification model. (iv) Calculate the resulting rRNA/DNA ratios explicitly, or create random subsamples of the rRNA and *rrn* gene pools, and use these surveys to calculate rRNA/DNA ratios. (v) Use the ratios to classify populations as active versus dormant. (vi) Calculate misclassification (FN and FP) rates. (vii) Repeat the simulation over 100 iterations.

To assess the accuracy of rRNA/DNA ratios for identifying population activity status (active versus dormant), misclassification rates were calculated. FP were populations that were known to be inactive (i.e., >50% of the cells were in the dead or dormant state) but had observed rRNA/DNA ratios of >1. Conversely, FN were populations that were known to be active (i.e., >50% of the cells were in the maintenance or growing state) but had rRNA/DNA ratios of <1. With these metrics, it was possible to quantify the effects of community structure, rRNA amplification model, and sampling depth on the accuracy of activity assessments derived from rRNA/DNA ratios.

The R code to perform these simulations is included in the supplemental material.

### Empirical sequence data.

Forest floor samples were collected in October 2011 at four sites (three replicates per site) over an approximately 500-km latitudinal gradient. Extraction of DNA and RNA and nucleic acid sequencing were described previously (22, 42). The primers employed to amplify bacterial 16S rRNA genes from DNA and cDNA were targeted to the V5 and V6 region of the gene as described by Claesson et al. (43). Sequencing was performed via 454 FLX Titanium at the Los Alamos National Laboratory. The resulting sequences were quality checked in the mothur software package v.1.27.0 (44) by using the PyroNoise algorithm (45). Potentially chimeric sequences were identified by using the mothur implementation of Perseus (46), and all prospective chimeras were removed from further analysis. The bacterial 16S rRNA sequences were aligned in mothur against the SILVA bacterial reference alignment (47), and OTUs were determined by clustering sequences by average neighbor clustering in mothur with a 97% sequence identity threshold.

The rRNA/DNA ratios of the empirical sequencing data were calculated after randomly subsampling paired DNA and rRNA libraries to the same number of reads to remove potential biases due to differences in sampling depth. The proportional abundance of each OTU was then determined (dividing the number of reads for each OTU by the number of reads in the sample). Only OTUs that were detected in both the *rrn* gene survey and the rRNA survey were employed to calculate rRNA/DNA ratios. The rRNA/DNA ratio of each OTU was determined by dividing the relative abundance of the OTU in the rRNA survey by its relative abundance in the *rrn* gene survey.

The taxonomic identities of OTUs in the empirical sequencing data were determined with the mothur-implemented Bayesian classifier (48) to compare OTU representative sequences against the SILVA reference database (47), release 123. Only taxonomic assignments with a confidence score of ≥70% are reported.

## Supplementary Material

Supplemental material
