# Supplementary material for: Simulated rRNA/DNA Ratios Show Potential To Misclassify Active Populations as Dormant
Source: Appl Environ Microbiol. 2017 May 17;83(11):e00696-17. doi: 10.1128/AEM.00696-17 (PMC5440720; doi:10.1128/AEM.00696-17)
Supplement: Supplemental material [file supp_83_11_e00696-17__index.html]

Simulated rRNA/DNA Ratios Show Potential To Misclassify Active Populations as Dormant — Supplemental material 

# Simulated rRNA/DNA Ratios Show Potential To Misclassify Active Populations as Dormant

## Supplemental material

- Supplemental file 1 -

  Published measurements of cellular ribosome abundance (Table S1) and R code for simulations.

  PDF, 175K
