# Supplementary material for: Simulated rRNA/DNA Ratios Show Potential To Misclassify Active Populations as Dormant
Source: Appl Environ Microbiol. 2017 May 17;83(11):e00696-17. doi: 10.1128/AEM.00696-17 (PMC5440720; doi:10.1128/AEM.00696-17)
Supplement: Supplemental material [file AEM.00696-17_zam999117861s1.pdf]

Table S1. Literature review of ribosome content per cell.

Organism	Method	Inactive[1]	Active/Growing	Reference
Escherichia coli	Direct imaging	-	30,000 – 70,000	2
Escherichia coli	Mass calculation	-	24,000	14
Escherichia coli	Estimate from synthesis	-	6,800	4
Escherichia coli	Estimate from synthesis	-	4,000 - 24,000	1
Escherichia coli	Estimate from synthesis	-	776 - 38,300	6
Escherichia coli	Estimate from synthesis	-	8,000 - 72,000	7
Bacillus subtilis	Fractionation	-	6,000	3
Rickettsia prowazekii	Mass calculation	-	1,500	14
Sphingomonas sp. RB2256	Mass calculation	200	2,000	8
Sulfate-reducing bacterium PT2	FISH fluorescence	-	8,000 – 35,000	15
Spiroplasma melliferum	Cryoelectron tomography	-	275	13
Mycobacterium fortuitum	Estimate from synthesis	-	4,000	10
Mycobacterium bovis BCG	Estimate from synthesis	-	687 - 3,880	4
Vibrio sp. CCUG 15956	Estimate from synthesis	8,000	20,000	9
Shewanella oneidensis MR-1	Proteomics	-	3,800 - 9,600	16
Pseudomonas stuzeri Zobell	RNA/DNA ratios	-	2,584	11
Thiomicrospira denitrificans	RNA/DNA qPCR	20-200	2,000	12
ARMAN archaea	3-D microscopy	-	92	5

[1]Ribosome content in stationary phase cells
[2]Ranges represent ribosome numbers between slow growing and fast growing cells

References used in Table S1.

1. Arfvidsson C, Wahlund KG (2003). Time-minimized determination of ribosome and tRNA levels in bacterial cells using flow field-flow fractionation. *Anal Biochem* **313:** 76-85.
2. Bakshi S, Siryaporn A, Goulian M, Weisshaar JC (2012). Superresolution imaging of ribosomes and RNA polymerase in live Escherichia coli cells. *Molecular Microbiology* **85:** 21-38.
3. Barrera A, Pan T (2004). Interaction of the Bacillus subtilis RNase P with the 30S ribosomal subunit. *RNA* **10:** 482-492.
4. Beste DJV, Peters J, Hooper T, Avignone-Rossa C, Bushell ME, McFadden J (2005). Compiling a molecular inventory for Mycobacterium bovis BCG at two growth rates: Evidence for growth rate-mediated regulation of ribosome biosynthesis and lipid metabolism. *Journal of Bacteriology* **187:** 1677-1684.

5. Comolli LR, Baker BJ, Downing KH, Siegerist CE, Banfield JF (2009). Three-dimensional analysis of the structure and ecology of a novel, ultra-small archaeon. *Isme J* **3:** 159-167.
6. Davis BD, Luger SM, Tai PC (1986). Role of ribosome degradation in the death of starved Escherichia coli cells. *J Bacteriol* **166:** 439-445.
7. Ehrenberg M, Bremer H, Dennis PP (2013). Medium-dependent control of the bacterial growth rate. *Biochimie* **95:** 643-658.
8. Fegatella F, Lim J, Kjelleberg S, Cavicchioli R (1998). Implications of rRNA operon copy number and ribosome content in the marine oligotrophic ultramicrobacterium Sphingomonas sp. strain RB2256. *Appl Environ Microb* **64:** 4433-4438.
9. Flardh K, Cohen PS, Kjelleberg S (1992). Ribosomes exist in large excess over the apparent demand for protein synthesis during carbon starvation in marine Vibrio sp. strain CCUG 15956. *J Bacteriol* **174:** 6780-6788.
10. Garcia MJ, Nunez MC, Cox RA (2010). Measurement of the Rates of Synthesis of Three Components of Ribosomes of Mycobacterium fortuitum: A Theoretical Approach to qRT-PCR Experimentation. *PLoS One* **5**.
11. Kerkhof L, Ward BB (1993). Comparison of Nucleic-Acid Hybridization and Fluorometry for Measurement of the Relationship between Rna/DNA Ratio and Growth-Rate in a Marine Bacterium. *Appl Environ Microb* **59:** 1303-1309.
12. Labrenz M, Brettar I, Christen R, Flavier S, Botel J, Hofle MG (2004). Development and application of a real-time PCR approach for quantification of uncultured bacteria in the central Baltic Sea. *Appl Environ Microb* **70:** 4971-4979.
13. Ortiz JO, Forster F, Kurner J, Linaroudis AA, Baumeister W (2006). Mapping 70S ribosomes in intact cells by cryoelectron tomography and pattern recognition. *J Struct Biol* **156:** 334-341.
14. Pang HL, Winkler HH (1994). The Concentrations of Stable Rna and Ribosomes in Rickettsia-Prowazekii. *Molecular Microbiology* **12:** 115-120.
15. Poulsen LK, Ballard G, Stahl DA (1993). Use of rRNA fluorescence insitu hybridization for measuring the activity of single cells in young and established biofilms. *Appl Environ Microb* **59:** 1354-1360.
16. Taylor RC, Robertson BJMW, Markillie LM, Serres MH, Linggi BE, Aldrich JT *et al* (2013). Changes in translational efficiency is a dominant regulatory mechanism in the environmental response of bacteria. *Integr Biol-Uk* **5:** 1393-1406

````
---
title: "R code for simulation of community sampling and derivation of rRNA:DNA ratios"
author: "Blaire Steven"
date: "January 9, 2017"
output: html_document
---

```{r setup, Echo=FALSE}
###########################################################################################
## This document contains functions used in the simulations of Steven et al. AEM 2017     #
## Following the functions, we give a brief demonstration of how the they can             #
## be used to visualize the results as presented in the manuscript.                       #
## Note that these are simulations and will differ between runs.  Outputs may differ      #
## from the manuscript or between independent implementations of the code.                #
##########################################################################################

###There are a total of four functions in this file, briefly summarized below:
## rand.abund.dist  - A function producing a vector from a uniform distribution.
##        Used to assign the community structure (abundance distribution of populations).
## gen.foldchange - Outputs simulation results, including rRNA:DNA ratios.
## iter_foldchange - Iterates the gen.foldchange function.
## seq_effort - A function that simulates incomplete sampling

########################Function gen.foldchange##################################
# The function gen.foldchange generates population-level information of simulated
# rRNA:DNA ratios. The function requires the three inputs listed below.
#
# 1. Specify the population size of the community (Comm.Size), set to 5000 populations
# for the present manuscript
# 2. The maximum number of rrn gene copies per cell (set at 1 for the present manuscript).
# 3. The community structure (defind by a log normal distribution with standard
#    deviation equal to LogNormSD), and the type of ribosome amplification model (amp.state;
#    choices are "low", "med","high", or "mix"). See manuscript for details.

#This function is primarily used within the iter_foldchange function, rather than
#singularly, although in a context where iterations are not necessary, it may be
#used alone.

# This function also requires the rand.abund.dist function (see above).

## The function gen.foldchange returns a list of values related to determining rRNA:DNA
#  ratios, listed below.

## ratio: The rRNA to DNA ratio determined by the simulation.
## met.state: Proportion of cells in each population that belong to each of the four
#  metabolic states (i.e. "Dead", "Dormant", "Maintenance", and "Growing" ).
## pop.dist: A distribution of rrn gene counts, drawn from a log normal distribution
#           with standard deviation of LogNormSD.
## rRNA.count: The rRNA count, based on metabolic state and ribosome amplification model.
## amp.designation: Tracks the ribosome amplification model used to determine rRNA counts.
#  This is employed to identify "low", "medium" and "high" ribosomal amplification
#  populations. Only useful in the "mixed" amplification model simulations, otherwise all
#  populations are the same as the input.


########################################################################################################
#######
###                                     Demonstration of Code
####
########################################################################################################
#######


# Randomly assign the proportion of cells to each of four metabolic states; each row has
# four columns, one for each metabolic state (i.e. "Dead", "Dormant", "Maitnance", "Growing",
# see manuscript)

gen.foldchange<-function(Comm.Size,Max_CopyNum,LogNormSD,amp.state){
  csize <- 1:Comm.Size
  met.state <- NULL
  for(i in seq(along=csize)) {
    met.state = rbind(met.state,rand.abund.dist(4))
  }
````
````

```
#Generate a distribution of ribosomal rrn gene (DNA) counts, called pop.dist, from a log normal
distribution with a standard deviation of LogNormSD.

distr<-(rlnorm(Comm.Size,0,LogNormSD))
  pop.dist<-rev(sort((distr/sum(distr))*100))

  #Adjust the distribution of DNA counts based on max potential rrn copy number.
  #In the present manuscript, maximum copy number was set to one. When maximum
  #copy number is set to 1, DNA.count will be equal to pop.dist.

copyNum.multiplier <- NULL
  for(i in seq(along=csize)){
    copyNum.multiplier <- rbind(copyNum.multiplier, sample(1:Max_CopyNum,1))
  }

  pop.mod<-pop.dist * copyNum.multiplier
  DNA.count <- 100*(pop.mod/(sum(pop.mod)))

  # Set ribosomal amplification, can vary between "low", "medium", "high, or "mix"
  # (see manuscript for details).

amp.designation <- NULL
  amp.multiplier <- NULL
  rint <- NULL
  if(amp.state == "low"){
    for(i in seq(along=csize)) {
      amp.multiplier <- rbind(amp.multiplier,c(1,100,200,500))
      amp.designation <- rbind(amp.designation, "low")
    }
  }else if(amp.state == "med"){
    for(i in seq(along=csize)) {
      amp.multiplier <- rbind(amp.multiplier,c(1,100,500,1000))
      amp.designation <- rbind(amp.designation, "med")
    }
  }else if(amp.state == "high"){
    for(i in seq(along=csize)) {
      amp.multiplier <- rbind(amp.multiplier,c(1,100,1000,10000))
      amp.designation <- rbind(amp.designation, "high")
    }
  }else if(amp.state == "mix"){
    for(i in seq(along=csize)){
      rint <- sample(1:3, 1)
      if(rint == 1){
        amp.multiplier <- rbind(amp.multiplier,c(1,100,200,500))
        amp.designation <- rbind(amp.designation, "low")
      }else if(rint == 2){
        amp.multiplier <- rbind(amp.multiplier,c(1,100,500,1000))
        amp.designation <- rbind(amp.designation, "med")
      }else if(rint == 3){
        amp.multiplier <- rbind(amp.multiplier,c(1,100,1000,10000))
        amp.designation <- rbind(amp.designation, "high")
      }
    }
  }else{
    stop()
  }

#Based on the metabolic state and the ribosome amplification model, generate the rRNA count.

rRNA<-rowSums(met.state * pop.dist * amp.multiplier)
  rRNA <- rRNA  * copyNum.multiplier
   rRNA.count<-100*(rRNA/sum(rRNA))

#Calculate the rRNA to rDNA ratio.
   ratio<-rRNA.count/DNA.count

  #Return the results as a named list.

  return(list(ratio=ratio,met.state=met.state,pop.dist=pop.dist,
              copyNum.multiplier=copyNum.multiplier,DNA.count=DNA.count,
              rRNA.count=rRNA.count,amp.designation=amp.designation,
              amp.multiplier=amp.multiplier))
```

```
}

######################## Function iter_foldchange ############################
# iter_foldchange iterates the process of simulating the rRNA:DNA ratio over the
# number of iterations specified via the iters argument.  Other than the iters argument,
# parameters required by this function are identical to those in gen.foldchange.

# Returns a list of iterated parameters related to simulating the rRNA:DNA ratios.
# The list contains the named objects below:
## i_ratio: The rRNA to DNA ratio determined by iterative, independent simulations.
## i_met.state: Proportion of a population's cells in each metabolic state.
## i_pop.dist: A distribution of rrn gene counts, drawn from a log normal distribution
#  with standard deviation of     LogNormSD
## i_copyNum.multiplier: The copy number used in the iterative sampling
## i_rRNA.count: The rRNA count, based on metabolic state and amplification model.
## i_amp.designation: The ribosome amplification for each cell.
## i_commstruc: The community structure used in simulations, equivalent to the input
#  parameter LogNormSD.
## i_active: Determines if a population is active or not based on the combined proportion of
# cells in maintenance or growing phases. If more than 50% of cells are in
# maintenance or growing phases, the population is set to TRUE, meaning active.


iter_foldchange<-function(Comm.Size, Max.CopyNum, LogNormSD, amp.state,iters){
  iter<-1:iters
  i_ratio<-NULL
  i_met.state<-NULL
  i_pop.dist<-NULL
  i_copyNum.multiplier<-NULL
  i_DNA.count<-NULL
  i_rRNA.count<-NULL
  i_amp.designation<-NULL
  iters_id <-NULL
  i_commstruc <- NULL
  i_active <- NULL
  for(i in seq(along=iter)) {
    tmp<-NULL
    tmp<-gen.foldchange(Comm.Size, Max.CopyNum, LogNormSD, amp.state)
    i_ratio<-c(i_ratio, tmp$ratio)
    i_active <-c(i_active,tmp[[2]][,3]+tmp[[2]][,4]>=50)
    i_met.state<-rbind(i_met.state,tmp$met.state)
    i_pop.dist<-c(i_pop.dist, tmp$pop.dist)
    i_copyNum.multiplier<-c(i_copyNum.multiplier, tmp$copyNum.multiplier)
    i_DNA.count<-c(i_DNA.count, tmp$DNA.count)
    i_rRNA.count<-c(i_rRNA.count, tmp$rRNA.count)
    i_amp.designation<-c(i_amp.designation, tmp$amp.designation)
    iters_id<-c(iters_id,rep(i,Comm.Size))
    i_commstruc <- c(i_commstruc,rep(LogNormSD,Comm.Size))
  }
  return(list(i_ratio=i_ratio, i_met.state=i_met.state,
              i_pop.dist=i_pop.dist, i_copyNum.multiplier=i_copyNum.multiplier,
              i_DNA.count=i_DNA.count, i_rRNA.count=i_rRNA.count,
              i_amp.designation=i_amp.designation,iters_id=iters_id,i_commstruc=
                i_commstruc,i_active=i_active))
}


###########################Function rand.abund.dist##############################
# This function is a helper function for gen.foldchange, producing a vector of
# equal to the length of the population distribution distribution that sums to 100.
# In the present manuscript, this function is used to generate the proportion of
# cells in each amplification model (only varies in mixed population simulations).

rand.abund.dist <- function(num) {x1<-(runif(num))
x2 <- (x1/sum(x1))*100
return(x2)}


########################### Function seq_effort #################################
# With  seq_effort, we mimic random sampling of natural communities by taking
# subsets of a community generated with the function gen.foldchange.

# seq_effort requires the following inputs:
```

```
# number of subsamples to draw (n),
# The inputs from the function gen.foldchange: DNA_df (for example, iterated DNA
# counts, e.g. df_med_1$i_DNA.count) and RNA_df (df_med_1$i_rRNA.count).
# seq_effort returns a list with two columns: sample ($sample),  the sample
# populations drawn from the original community, and sampleratio ($sampleratio),
# which returns the rRNA:DNA ratio of all populations drawn from the community.
#
# This function assumes the two inputs are the same order (that is,
# the first observation for each count corresponds to the same sample) and have the
# same number of observations.

seq_effort<-function(n, DNA_df, RNA_df){
  DNA_samp_temp <- sample(c(1:length(DNA_df)), size=n, prob=(DNA_df/100), replace=TRUE)
  DNA_samp_temp <- factor(DNA_samp_temp, 1:length(DNA_df))
  DNA_sample<-data.frame(table(DNA_samp_temp))
  cbind(DNA_sample,DNA_df) -> DNA_sample
  total_dna <- DNA_sample$Freq * DNA_df
  cbind(DNA_sample,total_dna)->DNA_sample
  RNA_samp_temp <- sample(c(1:length(RNA_df)), size=n, prob=(RNA_df/100),replace=TRUE)
  RNA_samp_temp <- factor(RNA_samp_temp, 1:length(RNA_df))
  RNA_sample<-data.frame(table(RNA_samp_temp))
  cbind(RNA_sample,RNA_df) -> RNA_sample
  total_rna <- RNA_sample$Freq * RNA_df
  cbind(RNA_sample,total_rna)->RNA_sample
  combined<-data.frame(DNA_sample, RNA_sample)
  seqEff_foldChange <- combined$total_rna/combined$total_dna
  samplesize <- rep(n,length(seqEff_foldChange))
  return(list(sample=combined, sampleratio=seqEff_foldChange,samplesize=samplesize))
}



####################### Manuscript examples #############################################
## We will now move on to worked examples that provide output similar to those used
## in the manuscript to give users a familiarity of the functions and their output.

###We load ggplot2 for plotting.
library(ggplot2)

# The first example here will plot three histograms, one per ribosome amplification model,
# similar to Figure 2 in the manuscript.

# We begin by estimating parameters for simulated populations. As in the manuscript,
# we set the population size to 5000, run across all three amplification states.  We also
# define community structure with a lognormal distribution with SD of 1, and we
# leave the copy number multiplier in all instances at 1.

# iter_foldchange takes five inputs: The population size, the maximum copy number
# (does not vary in the manuscript:set to 1), the standard deviation (used to simulate
# population structure), the amplification state of the input populations("low","med",
# "high", or "mix"), and the number of iterations.

df_low_sd1<-iter_foldchange(5000,1,1,"low",100)
df_med_sd1<-iter_foldchange(5000,1,1,"med",100)
df_high_sd1<-iter_foldchange(5000,1,1,"high",100)

#We can examine the output contents of each object using any of a number of commands,
#such as str()

str(df_low_sd1)

# Next we bind together the rRNA:DNA ratios from each amplifiation state to create
# one dataframe.  We then name the columns of the data frame, re-order the factors so
# they will appear in a logical manner in the plot, and finally plot three histograms,
# one for each amplification model.  We also add  labels to the facets.

cbind(data.frame(c(df_low_sd1$i_ratio,df_med_sd1$i_ratio,df_high_sd1$i_ratio)),
      data.frame(c(df_low_sd1$i_amp.designation,df_med_sd1$i_amp.designation,
                   df_high_sd1$i_amp.designation)))-> df_ratios

colnames(df_ratios) <- c("i_ratio","i_amp.designation")

df_ratios$i_amp.designation <- factor(df_ratios$i_amp.designation,
```

```
                                        levels=c("low","med","high"),
                                        labels=c("low","med","high"))

amp.states.plot <- c('low' = "Low ribosome amplification",
                     'med' = "Medium ribosome amplification",
                     'high' = "High ribosome amplification")


# To calculate the mean and max ratios in these populations and
# add this information to each histogram.  We will build a data frame based on
# this information.

aggregate(i_ratio~i_amp.designation,data=df_ratios,mean) -> means
  colnames(means) <- c("i_amp.designation","mean")
  paste("Mean RNA:DNA Ratio:", sprintf("%.2f", signif(means[,2],digits=3))) -> labs
aggregate(i_ratio~i_amp.designation,data=df_ratios,max) -> maxs
  colnames(maxs) <- c("i_amp.designation","max")
  paste("Max RNA:DNA Ratio:", sprintf("%.2f", signif(maxs[,2],digits=3))) -> labs2

paste(labs,"\n",labs2)->labs
cbind(data.frame(means$i_amp.designation),labs)->labs
colnames(labs)<-c("i_amp.designation","labs")


##### A code for plotting the data is presented below.

Figure.2 <- ggplot(df_ratios, aes_string(x=df_ratios$i_ratio)) +
            geom_histogram(binwidth=0.1) +
            facet_grid(~i_amp.designation, labeller=as_labeller(amp.states.plot)) +
            theme_bw() + xlab("rRNA:DNA ratios") +  ylab("Counts of rRNA:DNA Ratio bins") +
            geom_label(data=labs, hjust=0.5, aes(x=1.75, y=700, label=labs),
            colour="black", inherit.aes=FALSE, parse=FALSE,size=3) +
            theme(panel.grid.minor = element_blank())
Figure.2
ggsave(filename = "Figure_2.tiff")


# Below is an example that mimics part of the output represented in Table 1.
# Here we are interested in the false positive and false negative values
# from different amplification models and for different community structures.


#We use code similar to the above simulations and now iterate 100 times across
#three different community structures.

df_low_sd2<-iter_foldchange(50,1,2,"low",100)
df_med_sd2<-iter_foldchange(50,1,2,"med",100)
df_high_sd2<-iter_foldchange(50,1,2,"high",100)

df_low_sd1<-iter_foldchange(50,1,1,"low",100)
df_med_sd1<-iter_foldchange(50,1,1,"med",100)
df_high_sd1<-iter_foldchange(50,1,1,"high",100)

df_low_sd0<-iter_foldchange(50,1,0,"low",100)
df_med_sd0<-iter_foldchange(50,1,0,"med",100)
df_high_sd0<-iter_foldchange(50,1,0,"high",100)

# We can estimate how often false positives (FP) and false negatives (FN) occur
# when classifying activity based on rRNA:DNA ratios >1 by evaluating this ratio
# compared to whether a given population was set to active or not based on the
# proportion of cells in 'maintenance' or 'growing' (see manuscript for details).
#
# To examine if community structure alters the FN/FP rates, we begin by binding
# the above variables from each amplification state from a different community
# structure together.  We thus have three data frames that we will use to determine
# FN/FP values.


cbind(data.frame(c(df_low_sd1$i_ratio,df_low_sd0$i_ratio,df_low_sd2$i_ratio)),
      data.frame(c(df_low_sd1$i_amp.designation,df_low_sd0$i_amp.designation,
                   df_low_sd2$i_amp.designation)),
      data.frame(c(df_low_sd1$i_active,df_low_sd0$i_active,df_low_sd2$i_active)),
      data.frame(c(df_low_sd1$i_commstruc,df_low_sd0$i_commstruc,df_low_sd2$i_commstruc))
```

```
)-> df_low_fnfp

colnames(df_low_fnfp)<-c('i_raio','i_amp.designation','i_active','i_commstruc')

cbind(data.frame(c(df_med_sd1$i_ratio,df_med_sd0$i_ratio,df_med_sd2$i_ratio)),
      data.frame(c(df_med_sd1$i_amp.designation,df_med_sd0$i_amp.designation,
                   df_med_sd2$i_amp.designation)),
      data.frame(c(df_med_sd1$i_active,df_med_sd0$i_active,df_med_sd2$i_active)),
      data.frame(c(df_med_sd1$i_commstruc,df_med_sd0$i_commstruc,df_med_sd2$i_commstruc))
)-> df_med_fnfp

colnames(df_med_fnfp)<-c('i_raio','i_amp.designation','i_active','i_commstruc')

cbind(data.frame(c(df_high_sd1$i_ratio,df_high_sd0$i_ratio,df_high_sd2$i_ratio)),
      data.frame(c(df_high_sd1$i_amp.designation,df_high_sd0$i_amp.designation,
                   df_high_sd2$i_amp.designation)),
      data.frame(c(df_high_sd1$i_active,df_high_sd0$i_active,df_high_sd2$i_active)),
      data.frame(c(df_high_sd1$i_commstruc,df_high_sd0$i_commstruc,df_high_sd2$i_commstruc))
)-> df_high_fnfp

colnames(df_high_fnfp)<-c('i_raio','i_amp.designation','i_active','i_commstruc')

# Now we use these three dataframes to select false positive and false negative
# observations, then count the number of such observations and divide by our total
# sample size to get a percentage of false negatives and false positives.

# sample size should be adjusted if it varies from the default.
# Finally, we bind these observations together for to visualize FN/FP rates.

#False positives and negatives for the low amplfication state:
c(table(subset(df_low_fnfp$i_commstruc, df_low_fnfp[[1]]>1 &
                 df_low_fnfp$i_active==FALSE))/5000) -> lowfps
c(table(subset(df_low_fnfp$i_commstruc, df_low_fnfp[[1]]<1 &
                 df_low_fnfp$i_active==TRUE))/5000) -> lowfns

#False positives and negatives for the medium amplfication state:
c(table(subset(df_med_fnfp$i_commstruc, df_med_fnfp[[1]]>1 &
                 df_med_fnfp$i_active==FALSE))/5000) -> medfps
c(table(subset(df_med_fnfp$i_commstruc, df_med_fnfp[[1]]<1 &
                 df_med_fnfp$i_active==TRUE))/5000) -> medfns

#False positives and negatives for the high amplfication state:
c(table(subset(df_high_fnfp$i_commstruc, df_high_fnfp[[1]]>1 &
                 df_high_fnfp$i_active==FALSE))/5000) -> highfps
c(table(subset(df_high_fnfp$i_commstruc, df_high_fnfp[[1]]<1 &
                 df_high_fnfp$i_active==TRUE))/5000) -> highfns

#Bind the false positive/false negatives together.
data.frame(rbind(
  cbind(rep("low",3),c(0,1,2),lowfps,lowfns),
  cbind(rep("med",3),c(0,1,2),medfps,medfns),
  cbind(rep("high",3),c(0,1,2),highfps,highfns))) -> comm_res

#name the columns so they are easier to interpret
colnames(comm_res)<-c("amp.state","comm.str","FPs","FNs")

#Print the final output of the above binding operations.
comm_res


# The third example will plot three histograms that demonstrate how
# incomplete sampling can affect rRNA:DNA ratios, producing a figure
# similar to Figure 3 in the manuscript. Both this third example, and
# the example below, make use of the seq_effort function to partially
# sample rDNA and rRNA counts.

# We must first simulate the the data we plan to use in our subsampling simulations.
dfs_med_sd1<-iter_foldchange(5000,1,1,"med",1)

# note that the iterations are set to one, so that all samples are drawn from a
# single community
```

```
# seq_effort has three arguments: the number of samples, and then DNA and
# rRNA counts to subsample.  Here we use the samples from iter_foldchange as
# our input counts.

df_med_sd1_1x<-seq_effort(5000, dfs_med_sd1$i_DNA.count,
                          dfs_med_sd1$i_rRNA.count)
df_med_sd1_10x<-seq_effort(50000, dfs_med_sd1$i_DNA.count,
                           dfs_med_sd1$i_rRNA.count)
df_med_sd1_100x<-seq_effort(500000, dfs_med_sd1$i_DNA.count,
                            dfs_med_sd1$i_rRNA.count)
df_med_sd1_1000x<-seq_effort(5000000, dfs_med_sd1$i_DNA.count,
                              dfs_med_sd1$i_rRNA.count)


# As above, we bind the results together, then plot them.
# We also remove missing DNA and RNA observations (due to incomplete sampling)
# -indicated by 0s or INF in our data frame.
# If using values other than the default, make sure to adjust the division
# to the original population size!

cbind(data.frame(c(df_med_sd1_100x$sampleratio,
                   df_med_sd1_1000x$sampleratio,df_med_sd1_10000x$sampleratio)),
      data.frame(c(df_med_sd1_100x$samplesize,
                   df_med_sd1_1000x$samplesize,df_med_sd1_10000x$samplesize))
      )-> df_subratios

colnames(df_subratios) <- c("ratio","sample_size")
df_subratios$sample_size <- factor(df_subratios$sample_size)

#Remove any observations that either did not have RNA sampled (0) or DNA
#sampled (Inf).

df_subratios[df_subratios == 0] <- NA

df_subratios[df_subratios == 'Inf'] <- NA


#The above examples represent just one sampling iteration.
#To test if trends hold across multiple iterations we iterate the use of
#seq_effort for different values of 'n'.
#Due to the large dataframes produced, we do not store every iteration, but only
#summary statistics.  As long as output_means is not reset to NULL between
#runs, the loop will append the next set of summaries on to the object.
#Define the number of iterations and a null object, output_means, to store
#the results of the iteration:


iter = 1:100
output_means <- NULL

#next, the first sample size we will iterate (i.e. draw 5000 samples 100 times)
n=5000

# finally, run the loop below, which will populate output_means for an n of 5000
# Any change of n is run as a seperate loop.

for(i in seq(along=iter)){
  df_temp<-seq_effort(n, dfs_med_sd1$i_DNA.count,
                      dfs_med_sd1$i_rRNA.count)
  df_temp$sampleratio[df_temp$sampleratio== 0] <- NA

  df_temp$sampleratio[df_temp$sampleratio == 'Inf'] <- NA
  mean_temp<- mean(df_temp$sampleratio,na.rm=TRUE)
  max_temp <- max(df_temp$sampleratio,na.rm=TRUE)
  #Divide by 50 here because we are going to plot this as proportion
  #out of 100
  DNAsum_temp <- sum(df_temp$sample$total_dna != 0)/50
  RNAsum_temp <- sum(df_temp$sample$total_rna != 0)/50
  temp_sum <- cbind(mean_temp,max_temp,DNAsum_temp,RNAsum_temp,n)
  output_means <- rbind(output_means,temp_sum)
}
```

```
# To run  100 iterations of 50,000 random samples replace n=5000 with
# n=50000 above and rerun the loop.
# This can be repeated for multiple sample sizes.

# NOTE: EACH NEW RUN OF THE LOOP IS ADDED TO temp_sum. THERFORE VERLY
# LARGE SAMPLE SIZES OR LARGE NUMBERS OF REPAETED LOOPS WILL RESULT IN
# VERY LARGE FILE SIZES AND MAY RESULT IN MEMORY ERRORS.



#We now generate summary values from the above iterations, and create objects to store
#these iterated values.  Then, we will create a labeling object from these values to
#include on a plot.

aggregate(mean_temp~n,data=output_means,mean) -> means
aggregate(max_temp~n,data=output_means,max) -> maxs
aggregate(max_temp~n,data=output_means,mean) -> meanmaxs
aggregate(DNAsum_temp~n,data=output_means,mean) -> dnasums
aggregate(RNAsum_temp~n,data=output_means,mean) -> rnasums

#Now combine each of these into a table:
sample_size <- c("1X","10X","100X","10000X")
iter_table <- cbind(sample_size,means,maxs[,2],meanmaxs[,2],dnasums[,2],rnasums[,2])
colnames(iter_table) <- c("sample size","n","mean ratio","max ratio","mean max ratio",
                          "mean prop. dna sampled","mean prop. rna sampled")
print(iter_table)


#Create labels for the figure, if desired:
paste("Iterated Mean RNA:DNA Ratio:", sprintf("%.2f", round(means[,2],digits=2))) -> labs1
paste("Iterated Max RNA:DNA Ratio:", sprintf("%.2f", round(maxs[,2],digits=2))) -> labs2
paste("Iterated Mean Prop. RNA Sampled:",
      sprintf("%.2f", round(rnasums[,2],digits=2))) -> labs3
paste("Iterated Mean Prop. DNA Sampled:",
      sprintf("%.2f", round(dnasums[,2],digits=2))) -> labs4
paste(labs1,"\n",labs2,"\n",labs3,"\n",labs4)->labs
cbind(data.frame(means$n),labs)->labs
colnames(labs)<-c("sample_size","labs")




# As mentioned previously, because of the size of vectors produced during
# iteration,values of every rRNA:DNA ratio were not stored. Note that, because
# this is a single iteration being plotted, it is possible the maximum ratio
# (derived from 100 iterations)  may exceed any one iteration's maximum value.


sample.size.plot <- c('5000' = "5,000 samples", '50000' = "50,000 samples",
                      '5e+05' = "500,000 samples",'5e+06'="5,000,000 samples")

Figure.3 <- ggplot(df_subratios, aes_string(x=df_subratios$ratio)) +
  geom_histogram(aes(y=(..count../sum(..count..))*100),binwidth=0.1) +
  facet_wrap(~sample_size,shrink=T, scales = "free_y", labeller=as_labeller(sample.size.plot)) +
  geom_vline(xintercept = 1.0, linetype="dashed", colour="lightgray", size= 0.5) +
  theme_bw() + xlab("rRNA:DNA ratios") +  ylab("Percent of rRNA:DNA Ratios") +
  geom_label(data=labs, hjust=0, aes(x=3.2, y=c(0.4, 1.25, 1.25, 1.25), label=labs),
             colour="black", inherit.aes=FALSE, parse=FALSE, size=1.5) +
  theme(panel.grid.minor = element_blank())

Figure.3

ggsave(filename = "Figure_3.tiff")


# Next we will demonstrate how to assess the rate of false positives and false
# negatives, given different sample sizes and different ribosomal amplification
# models. We will also use 100 independent resamplings for each amplification model
# and sample size, finding the average and standard deviation of all groups.

######### NOTE: THE FOLLOWING CODE MAY TAKE A VERY LONG TIME TO RUN For Large Samples####
# ##Because of this, all lines below have been commented out to prevent incidental
# ##running of the loops.
```

```
# #Set the number of times we will repeat the re-sampling

# con<-1:100
# fpfn<-NULL
#
# # #For 50,000 samples, "low" amplification:
# for(i in seq(along=con)) {
# #
# # # Create several temporary variables to hold temporary results from a given loop:
# fp_temp <- NULL
# fn_temp <- NULL
# # #
# # # #Create the data frame from which we draw DNA and RNA counts during resampling:
# df_tmp<-iter_foldchange(5000, 1, 1, "low", 1)
# df_tmp_seq<-seq_effort(50000, df_tmp$i_DNA.count, df_tmp$i_rRNA.count)
# # #
# # # # count False negatives then divide by total community size (5000 members)
# fp_temp<-rbind(fp_temp, (length(subset(df_tmp_seq$sampleratio,
#                                    df_tmp$i_active==FALSE & df_tmp_seq$sampleratio > 1))/5000))
# # #
# # # # count False positives then divide by community size (5000 members).
#      fn_temp<-rbind(fn_temp, (length(subset(df_tmp_seq$sampleratio,
#                                    df_tmp$i_active==TRUE & df_tmp_seq$sampleratio < 1))/5000))
# # # #Bind fn and fps together
#      fps_temp <- data.frame(cbind(fn_temp,fp_temp))
# # #
# # #
# # #  #add the amplification designation and the sample size
#    samples <- 50000
#    fps_temp <- cbind(fps_temp,df_tmp$i_amp.designation[1],samples)
# # # # # return fpfn with false classification information for a single run.
#   fpfn <- rbind(fpfn,fps_temp)
#    }
#
# # # ##### Differnt sample sizes are listed below to replicate Table 2 in the manuscript
# # #
# # # # #For 50,000 samples, "medium" amplification:
# for(i in seq(along=con)) {
# fp_temp <- NULL
# fn_temp <- NULL
# df_tmp<-iter_foldchange(5000, 1, 1, "med", 1)
# df_tmp_seq<-seq_effort(50000, df_tmp$i_DNA.count, df_tmp$i_rRNA.count)
# fp_temp<-rbind(fp_temp, (length(subset(df_tmp_seq$sampleratio,
#                                        df_tmp$i_active==FALSE & df_tmp_seq$sampleratio > 1))/5000))
#    fn_temp<-rbind(fn_temp, (length(subset(df_tmp_seq$sampleratio,
#                                        df_tmp$i_active==TRUE & df_tmp_seq$sampleratio < 1))/5000))
#    fps_temp <- data.frame(cbind(fn_temp,fp_temp))
#    samples <- 50000
#    fps_temp <- cbind(fps_temp,df_tmp$i_amp.designation[1],samples)
#    fpfn <- rbind(fpfn,fps_temp)
# }
#
# #
# # # # #For 50,000 samples, "high" amplification:
# for(i in seq(along=con)) {
# fp_temp <- NULL
# fn_temp <- NULL
# df_tmp<-iter_foldchange(5000, 1, 1, "high", 1)
# df_tmp_seq<-seq_effort(50000, df_tmp$i_DNA.count, df_tmp$i_rRNA.count)
# fp_temp<-rbind(fp_temp, (length(subset(df_tmp_seq$sampleratio,
#                                        df_tmp$i_active==FALSE & df_tmp_seq$sampleratio > 1))/5000))
#    fn_temp<-rbind(fn_temp, (length(subset(df_tmp_seq$sampleratio,
#                                        df_tmp$i_active==TRUE & df_tmp_seq$sampleratio < 1))/5000))
#    fps_temp <- data.frame(cbind(fn_temp,fp_temp))
#    samples <- 50000
#    fps_temp <- cbind(fps_temp,df_tmp$i_amp.designation[1],samples)
#    fpfn <- rbind(fpfn,fps_temp)
#  }
#
# # # # #For 500,000 samples of sequencing effort, "low" amplification:
# for(i in seq(along=con)) {
# fp_temp <- NULL
```

```
# fn_temp <- NULL
# df_tmp<-iter_foldchange(5000, 1, 1, "low", 1)
# df_tmp_seq<-seq_effort(500000, df_tmp$i_DNA.count, df_tmp$i_rRNA.count)
# fp_temp<-rbind(fp_temp, (length(subset(df_tmp_seq$sampleratio,
#                                          df_tmp$i_active==FALSE & df_tmp_seq$sampleratio > 1))/5000))
#    fn_temp<-rbind(fn_temp, (length(subset(df_tmp_seq$sampleratio,
#                                          df_tmp$i_active==TRUE & df_tmp_seq$sampleratio < 1))/5000))
#    fps_temp <- data.frame(cbind(fn_temp,fp_temp))
#    samples <- 500000
#    fps_temp <- cbind(fps_temp,df_tmp$i_amp.designation[1],samples)
#    fpfn <- rbind(fpfn,fps_temp)
# }
#
# # # #For 500,000 samples of sequencing effort, "medium" amplification:
#  for(i in seq(along=con)) {
#    fp_temp <- NULL
#    fn_temp <- NULL
#    df_tmp<-iter_foldchange(5000, 1, 1, "med", 1)
#    df_tmp_seq<-seq_effort(500000, df_tmp$i_DNA.count, df_tmp$i_rRNA.count)
#    fp_temp<-rbind(fp_temp, (length(subset(df_tmp_seq$sampleratio,
#                                          df_tmp$i_active==FALSE & df_tmp_seq$sampleratio > 1))/5000))
#    fn_temp<-rbind(fn_temp, (length(subset(df_tmp_seq$sampleratio,
#                                          df_tmp$i_active==TRUE & df_tmp_seq$sampleratio < 1))/5000))
#    fps_temp <- data.frame(cbind(fn_temp,fp_temp))
#    samples <- 500000
#    fps_temp <- cbind(fps_temp,df_tmp$i_amp.designation[1],samples)
#    fpfn <- rbind(fpfn,fps_temp)
#  }
#
# # # # #For 500,000 samples of sequencing effort, "high" amplification:
#   for(i in seq(along=con)) {
#    fp_temp <- NULL
#    fn_temp <- NULL
#    df_tmp<-iter_foldchange(5000, 1, 1, "high", 1)
#    df_tmp_seq<-seq_effort(500000, df_tmp$i_DNA.count, df_tmp$i_rRNA.count)
#    fp_temp<-rbind(fp_temp, (length(subset(df_tmp_seq$sampleratio,
#                                          df_tmp$i_active==FALSE & df_tmp_seq$sampleratio > 1))/5000))
#    fn_temp<-rbind(fn_temp, (length(subset(df_tmp_seq$sampleratio,
#                                          df_tmp$i_active==TRUE & df_tmp_seq$sampleratio < 1))/5000))
#    fps_temp <- data.frame(cbind(fn_temp,fp_temp))
#    samples <- 500000
#    fps_temp <- cbind(fps_temp,df_tmp$i_amp.designation[1],samples)
#    fpfn <- rbind(fpfn,fps_temp)
#  }
#
# # # # #For 5,000,000 samples of sequencing effort, "low" amplification:
#  for(i in seq(along=con)) {
#    fp_temp <- NULL
#    fn_temp <- NULL
#    df_tmp<-iter_foldchange(5000, 1, 1, "low", 1)
#    df_tmp_seq<-seq_effort(5000000, df_tmp$i_DNA.count, df_tmp$i_rRNA.count)
#    fp_temp<-rbind(fp_temp, (length(subset(df_tmp_seq$sampleratio,
#                                          df_tmp$i_active==FALSE & df_tmp_seq$sampleratio > 1))/5000))
#    fn_temp<-rbind(fn_temp, (length(subset(df_tmp_seq$sampleratio,
#                                          df_tmp$i_active==TRUE & df_tmp_seq$sampleratio < 1))/5000))
#    fps_temp <- data.frame(cbind(fn_temp,fp_temp))
#    samples <- 5000000
#    fps_temp <- cbind(fps_temp,df_tmp$i_amp.designation[1],samples)
#    fpfn <- rbind(fpfn,fps_temp)
#  }
# # #
# # # # #For 5,000,000 samples of sequencing effort, "medium" amplification:
#  for(i in seq(along=con)) {
#    fp_temp <- NULL
#    fn_temp <- NULL
#    df_tmp<-iter_foldchange(5000, 1, 1, "med", 1)
#    df_tmp_seq<-seq_effort(5000000, df_tmp$i_DNA.count, df_tmp$i_rRNA.count)
#    fp_temp<-rbind(fp_temp, (length(subset(df_tmp_seq$sampleratio,
#                                          df_tmp$i_active==FALSE & df_tmp_seq$sampleratio > 1))/5000))
#    fn_temp<-rbind(fn_temp, (length(subset(df_tmp_seq$sampleratio,
#                                          df_tmp$i_active==TRUE & df_tmp_seq$sampleratio < 1))/5000))
#    fps_temp <- data.frame(cbind(fn_temp,fp_temp))
#    samples <- 5000000
```

```
#    fps_temp <- cbind(fps_temp,df_tmp$i_amp.designation[1],samples)
#    fpfn <- rbind(fpfn,fps_temp)
#  }
# # #
# # # # #For 5,000,000 samples of sequencing effort, "high" amplification:
#  for(i in seq(along=con)) {
#    fp_temp <- NULL
#    fn_temp <- NULL
#    df_tmp<-iter_foldchange(5000, 1, 1, "high", 1)
#    df_tmp_seq<-seq_effort(5000000, df_tmp$i_DNA.count, df_tmp$i_rRNA.count)
#    fp_temp<-rbind(fp_temp, (length(subset(df_tmp_seq$sampleratio,
#                                      df_tmp$i_active==FALSE & df_tmp_seq$sampleratio > 1))/5000))
#    fn_temp<-rbind(fn_temp, (length(subset(df_tmp_seq$sampleratio,
#                                      df_tmp$i_active==TRUE & df_tmp_seq$sampleratio < 1))/5000))
#    fps_temp <- data.frame(cbind(fn_temp,fp_temp))
#    samples <- 5000000
#    fps_temp <- cbind(fps_temp,df_tmp$i_amp.designation[1],samples)
#    fpfn <- rbind(fpfn,fps_temp)
#  }
#
# # # # #For 5,000,000 samples of sequencing effort, "low" amplification:
#  for(i in seq(along=con)) {
#    fp_temp <- NULL
#    fn_temp <- NULL
#    df_tmp<-iter_foldchange(5000, 1, 1, "low", 1)
#    df_tmp_seq<-seq_effort(50000000, df_tmp$i_DNA.count, df_tmp$i_rRNA.count)
#    fp_temp<-rbind(fp_temp, (length(subset(df_tmp_seq$sampleratio,
#                                      df_tmp$i_active==FALSE & df_tmp_seq$sampleratio > 1))/5000))
#    fn_temp<-rbind(fn_temp, (length(subset(df_tmp_seq$sampleratio,
#                                      df_tmp$i_active==TRUE & df_tmp_seq$sampleratio < 1))/5000))
#    fps_temp <- data.frame(cbind(fn_temp,fp_temp))
#    samples <- 50000000
#    fps_temp <- cbind(fps_temp,df_tmp$i_amp.designation[1],samples)
#    fpfn <- rbind(fpfn,fps_temp)
#  }
# # #
# # # # #For 50,000,000 samples of sequencing effort, "medium" amplification:
#  for(i in seq(along=con)) {
#    fp_temp <- NULL
#    fn_temp <- NULL
#    df_tmp<-iter_foldchange(5000, 1, 1, "med", 1)
#    df_tmp_seq<-seq_effort(50000000, df_tmp$i_DNA.count, df_tmp$i_rRNA.count)
#    fp_temp<-rbind(fp_temp, (length(subset(df_tmp_seq$sampleratio,
#                                      df_tmp$i_active==FALSE & df_tmp_seq$sampleratio > 1))/5000))
#    fn_temp<-rbind(fn_temp, (length(subset(df_tmp_seq$sampleratio,
#                                      df_tmp$i_active==TRUE & df_tmp_seq$sampleratio < 1))/5000))
#    fps_temp <- data.frame(cbind(fn_temp,fp_temp))
#    samples <- 50000000
#    fps_temp <- cbind(fps_temp,df_tmp$i_amp.designation[1],samples)
#    fpfn <- rbind(fpfn,fps_temp)
#  }
# # #
# # # # #For 50,000,000 samples of sequencing effort, "high" amplification:
#  for(i in seq(along=con)) {
#    fp_temp <- NULL
#    fn_temp <- NULL
#    df_tmp<-iter_foldchange(5000, 1, 1, "high", 1)
#    df_tmp_seq<-seq_effort(50000000, df_tmp$i_DNA.count, df_tmp$i_rRNA.count)
#    fp_temp<-rbind(fp_temp, (length(subset(df_tmp_seq$sampleratio,
#                                      df_tmp$i_active==FALSE & df_tmp_seq$sampleratio > 1))/5000))
#    fn_temp<-rbind(fn_temp, (length(subset(df_tmp_seq$sampleratio,
#                                      df_tmp$i_active==TRUE & df_tmp_seq$sampleratio < 1))/5000))
#    fps_temp <- data.frame(cbind(fn_temp,fp_temp))
#    samples <- 50000000
#    fps_temp <- cbind(fps_temp,df_tmp$i_amp.designation[1],samples)
#    fpfn <- rbind(fpfn,fps_temp)
#  }
#
# ##Below code requires that the above loops have been run.
# #Name the columns of the fpfn dataframe.
# colnames(fpfn) <- c("fn","fp","amp.state","samples")
#
# #Use the aggregate function to display mean percents:
```

~~~
# aggregate(cbind(fp,fn)~amp.state+samples,data=fpfn,mean)
#
# #Alternatively, standard deviations could be displayed:
# aggregate(cbind(fp,fn)~amp.state+samples,data=fpfn,sd)


```
~~~
